# Fitness dynamics within a poplar hybrid zone: I. Prezygotic and postzygotic barriers impacting a native poplar hybrid stand

**DOI:** 10.1002/ece3.1029

**Published:** 2014-04-03

**Authors:** Amanda D Roe, Chris J K MacQuarrie, Marie-Claude Gros-Louis, J Dale Simpson, Josyanne Lamarche, Tannis Beardmore, Stacey L Thompson, Philippe Tanguay, Nathalie Isabel

**Affiliations:** 1 Natural Resources Canada, Canadian Forest Service, Laurentian Forestry CentreQuébec, Québec, Canada; 2 Natural Resources Canada, Canadian Forestry Centre, Great Lakes Forestry CentreSault Ste. Marie, Ontario, Canada; 3 Natural Resources Canada, Canadian Forest Service, Atlantic Forestry CentreFredericton, New Brunswick, Canada; 4 Umeå University, Ecology and Environmental Sciences, Umeå Plant Science CentreUmeå, Sweden

**Keywords:** Hybrid fitness, hybridization, introgression, postzygotic fitness, reproductive fitness, SNPs

## Abstract

Hybridization and introgression are pervasive evolutionary phenomena that provide insight into the selective forces that maintain species boundaries, permit gene flow, and control the direction of evolutionary change. Poplar trees (*Populus* L.) are well known for their ability to form viable hybrids and maintain their distinct species boundaries despite this interspecific gene flow. We sought to quantify the hybridization dynamics and postzygotic fitness within a hybrid stand of balsam poplar (*Populus balsamifera* L.), eastern cottonwood (*P. deltoides* Marsh.), and their natural hybrids to gain insight into the barriers maintaining this stable hybrid zone. We observed asymmetrical hybrid formation with *P. deltoides* acting as the seed parent, but with subsequent introgression biased toward *P. balsamifera*. Native hybrids expressed fitness traits intermediate to the parental species and were not universally unfit. That said, native hybrid seedlings were absent from the seedling population, which may indicate additional selective pressures controlling their recruitment. It is imperative that we understand the selective forces maintaining this native hybrid zone in order to quantify the impact of exotic poplar hybrids on this native system.

## Introduction

Interspecific hybridization is a complex evolutionary phenomenon: two genetically distinct populations interbreed and produce admixed offspring (Abbott et al. 2013). When the biologic species concept was postulated by Mayr (1942), hybridization was viewed as a rare phenomenon – “good” species did not form hybrids (but see Anderson 1949). Hybridization and introgression are now recognized for their integral role in evolutionary diversification, adaptation, and biodiversity, which is a substantial paradigm shift within the evolutionary and ecological scientific communities (Abbott et al. 2013). Hybridization is prevalent among both plants (Arnold 1997) and animals (Mallet 2005) and is an important aspect of evolution with highly variable outcomes. Interspecific hybrids may be evolutionary dead ends (infertile and sterile) or may experience hybrid vigor and exhibit an adaptive advantage over the parental species (Schweitzer et al. 2002; Arnold and Martin 2010; Whitney et al. 2010). Hybridization may be an important source of new genetic variation (Butlin and Ritchie 2013) and can act as a selective filter, allowing only the introgression of adaptive genes while maintaining distinct species boundaries (Martinsen et al. 2001). In extreme cases, hybrid offspring may form a novel hybrid species (Anderson and Stebbins 1954; Rieseberg 1997). Conversely, hybridization and introgression can lead to the collapse of species boundaries (Rhymer and Simberloff 1996; Seehausen 2006) and negatively impact local populations when occurring in conjunction with invasive exotics (Schierenbeck and Ellstrand 2009) or anthropogenic disturbance (Seehausen 1997; Lamont et al. 2003; Vonlanthen et al. 2012).

Two distinct phases occur within natural hybrid zones: hybridization and subsequent introgression. Hybridization is the initial cross (*F*_1_) between two distinct parental species, while introgression occurs in successive steps where hybrids backcross with a parental species and interspecific genetic material moves into the parental genetic background. Initial formation of the *F*_1_ hybrid is controlled by both prezygotic and postzygotic barriers (Rieseberg and Carney 1998; Tiffin et al. 2001; Coyne and Orr 2004). Following hybrid formation, the fitness of the hybrid is determined by intrinsic (endogenous) and extrinsic (exogenous) factors (Stebbins 1950; Barton 2001; Burke and Arnold 2001; Arnold and Martin 2010). Endogenous selection is dependent on the intrinsic qualities of the hybrid organism and is independent of the environment (Barton and Hewitt 1985). An inherent loss of fitness may result from genetic incompatibilities (e.g., Bateson–Dobzhansky–Muller incompatibilities), negative epistasis, disruption of co-adapted gene complexes, or deleterious gene interactions, while high fitness may result from heterosis, transgressive segregation, or selective filtering of adaptive gene regions (Dobzhansky 1970; Burke and Arnold 2001; Martinsen et al. 2001; Tiffin et al. 2001; Rieseberg et al. 2003). When hybrid fitness is controlled by exogenous selection, there is an interaction between the hybrid genotype and its environment. Hybrid fitness is dependent on the environmental conditions experienced by the hybrid individual and may exceed that of parental species in novel habitats (Arnold 1997; Arnold and Martin 2010). The relative importance of endogenous and exogenous selection to hybrid zone dynamics has been contentious (Barton 2001), although both likely contribute to overall hybrid zone dynamics (Abbott et al. 2013).

Some of the most well-known hybrid systems involve complexes of forest trees including *Quercus* (e.g., Petit et al. 2003; Lepais and Gerber 2011), *Eucalyptus* (e.g., Field et al. 2011), *Picea* (e.g., Perron and Bousquet 1997), *Pinus* (e.g., Cullingham et al. 2012), and *Populus*. Poplar trees (*Populus* L.) are dioecious and well known for their ability to form interspecific hybrids. For this reason, they have been studied extensively (e.g., Eckenwalder 1984; Keim et al. 1989; Stettler et al. 1996a; Dickmann et al. 2001; Floate 2004; Vanden Broeck et al. 2005; Meirmans et al. 2010). Identification of hybrid poplars is possible using morphology (Floate 2004), but molecular markers (Smulders et al. 2001; Meirmans et al. 2007; Talbot et al. 2011; Isabel et al. 2013) have improved the detection of complex and advanced-generation hybrids. In eastern Canada, *P. balsamifera* L. and *P. deltoides* Marsh. are broadly sympatric, forming a stable hybrid zone in which both *F*_1_ and advanced-generation hybrids occur, and asymmetric introgression has been documented (Eckenwalder 1984; Floate 2004; Hamzeh et al. 2007; Thompson et al. 2010; LeBoldus et al. 2013). Rates of hybridization differ greatly between sites (Meirmans et al. 2010; Thompson et al. 2010; Talbot et al. 2012) and are dependent on population size (Meirmans et al. 2009, 2010). These patterns are consistent with those observed in other poplar hybrid zones in North America (e.g., Keim et al. 1989; Martinsen et al. 2001; Floate 2004) and Europe (Lexer et al. 2010; Vanden Broeck et al. 2012). In addition to these two native species, many exotic varieties of poplar have been planted as horticultural trees and used in commercial applications, such as biomass production (Richardson et al. 2007; Hinchee et al. 2009). Given the complex nature of the poplar community in eastern Canada, we needed to understand the fitness dynamics of the native *P. balsamifera* and *P. deltoides* hybrid zone before we could identify differences between native and exotic hybrid formation and then assess the potential impacts of exotic hybrids on this native system, which is addressed in our companion paper (Roe et al. 2014).

## Objectives

The impacts of hybridization and introgression on the evolutionary trajectory of species are dependent on the frequency of hybridization, the fertility of hybrid offspring, and the relative fitness of hybrid offspring and parental species. Many studies have examined hybridization in poplars, although few have examined the fitness of hybrids within their natural environment (but see Schweitzer et al. 2002). We examined the hybridization dynamics and fitness of poplars in a naturally regenerated stand of *Populus* at the Base de plein-air de Sainte-Foy (BPSF) in Quebec City, QC, Canada. Thompson et al. (2010) detected interspecific hybrids at BPSF in a survey of natural populations of *P. balsamifera* and *P. deltoides* throughout their zone of contact in eastern Canada. To further explore the dynamics of hybridization at BPSF, we screened adult trees, seeds, and naturally regenerated seedlings with a diagnostic single-nucleotide polymorphism (SNP) array to quantify rates of hybridization and the realized rate of introgression among native poplars. We also related four endogenous fitness traits (seed quantity, seed quality, seed germination, and disease susceptibility) to hybrid status to assess the relative fitness of pure parental species and natural hybrids. We used these fitness traits to identify individual components of postzygotic reproductive isolation among *P. balsamifera* and *P. deltoides* and to quantify the barriers that help to maintain the stable hybrid zone between these two distinct forest tree species.

## Methods

### Study site

**Figure 1:**
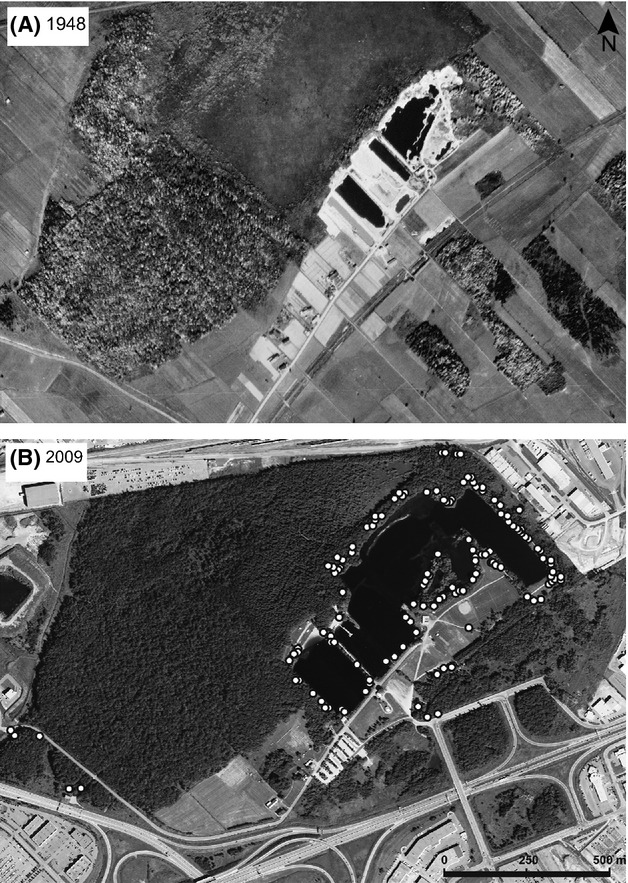
Aerial imagery of the Base de plein-air de Sainte-Foy collection site. (A) Site in 1948 during gravel extraction. (B) Site in 2009 showing regeneration with the location of sampled adult trees.

The BPSF is a 136 hectare recreational park located in Quebec City, Quebec, Canada, with an artificial lake surrounded by a mix of forest, managed grassland, and peatland (Fig. 1). The forest is a mix of poplar, maple (*Acer* spp.), and larch (*Larix laricina*). This site was developed as a gravel pit approximately 60 years ago (Fig. 1A). Following gravel extraction, vegetation was allowed to naturally recolonize the exposed mineral soil.

### Study system

The genus *Populus* is divided into six sections. *P. balsamifera* belongs to the Tacamahaca section, while *P. deltoides* is placed in section Aigeiros (Eckenwalder 1996). Hybrid formation is usually limited to species belonging to the same section. However, hybridization is common between *P. balsamifera* and *P. deltoides*, despite their evolutionary divergence (Eckenwalder 1984, 1996). Both species have broad geographic ranges, with *P. balsamifera* found throughout the boreal forest from Alaska to Newfoundland and *P. deltoides* found throughout eastern North America, from the Midwest to the Atlantic and extending from the Gulf of Mexico to southern Quebec. *P. balsamifera* and *P. deltoides* overlap along a relatively narrow region in the southeastern and northeastern edges of their ranges, respectively. The BPSF is located within this zone of overlap between *P. balsamifera* and *P. deltoides* where these species form a zone of hybridization. The BPSF is also an urban site and is in close proximity to a range of exotic poplar cultivars used as horticultural stock in urban landscaping. In addition to native hybrids, exotic hybrids, containing *P. nigra* and *P. maximowiczii* alleles (Thompson et al. 2010), were previously detected at BPSF.

### Sampling

#### Adult trees

Thompson et al. (2010) surveyed 15 reproductively mature trees in 2007. We sampled leaves from an additional 127 trees for a total of 142 adult trees. To minimize the chances of sampling clones, we selected trees that were at least 10 m apart and sampled the largest tree in the cohort. We collected leaves in paper envelopes and allowed them to dry at room temperature prior to genotyping. We recorded tree location, gender, age class, diameter at breast height (DBH), and height. Age class was determined by counting the number of verticils (i.e., whorls) on each reproductively mature tree and used to assign individuals to one of four age classes: <20, 20–40, 40–60, and >60 years of age.

#### Seeds

We sampled ripened catkins using a combination of pole pruners, climbing, and with the aid of a bucket truck due to the height of the available trees. Catkins were sampled in 2009–2011 from a total of 14 female trees (three *P. balsamifera*, four *P. deltoides*, seven native hybrids) prior to dehiscence (June 15 and 29). From each seed lot, we selected between 46 and 94 seeds per female tree (mean 48 ± 11) for genotyping. We placed catkins from each tree into separate paper bags and transported them to the laboratory where they were stored in a drying chamber for 3 days at 25°C until seeds dehisced. The seeds were then extracted from the capsule and frozen at −20°C prior to genotyping.

#### Regenerated seedlings

We surveyed for newly established poplar seedlings at seven sites at BPSF. Seedlings were sampled from five circular plots (3 m in diameter) and two linear transects that ran along walking paths (85 m and 125 m long, respectively). All poplar seedlings <4 years old (or <4 cm in diameter) from our seven sample sites were collected and preserved for genotyping. Seedlings were examined to ensure that there were no connections to parental root systems and that each individual was the product of sexual reproduction.

### Genotyping

#### DNA extraction

DNA was extracted from leaves and seeds with the Nucleospin 96 plant kit (Macherey-Nagel, Bethlehem, PA) following the protocols described in Isabel et al. (2013). DNA from seedlings was extracted with MagAttract 96 DNA Plant Core Kit (Qiagen, Mississauga, ON) according to the manufacturer's instructions.

#### SNP genotyping

The samples were genotyped with the Sequenom iPlex Gold high-throughput genotyping technology (Sequenom, Cambridge, MA) at the McGill University and Genome Quebec Innovation Centre (Montreal, QC) using their internal protocols. We typed each sample using an array of 36 diagnostic SNP markers (Isabel et al. 2013). This SNP array allowed simultaneous detection of eight poplar species: *P. angustifolia* James, *P. balsamifera*, *P. deltoides*, *P. fremontii* Watson, *P. laurifolia* Ledeb., *P. maximowiczii* Henry, *P. nigra* L., and *P. trichocarpa* Torr. and Gray (Isabel et al. 2013; Table S4 therein), which was required for this experiment given that poplars bearing exotic components have been previously detected at the BPSF (Thompson et al. 2010). The SNPs were located in 28 gene regions distributed across 18 chromosomes and were all unlinked. A complete SNP data file for all sampled individuals has been deposited in the Dryad data repository (http://doi:10.5061/dryad.63fr7). We also screened adult trees with two panels of intraspecific SNPs to detect potential clones in the sample populations. The *P. balsamifera* panel contained 35 intraspecific SNP markers, and the *P. deltoides* panel contained 33 intraspecific SNP markers (N. Isabel, unpubl. data).

#### Chloroplast haplotyping

We amplified the chloroplastic DNA with primers (c–d) (Taberlet et al. 1991) to identify the maternal lineage of each hybrid adult. DNA amplified following protocols described by Gros-Louis et al. (2005). Chloroplast DNA fragments were digested with the restriction enzyme HpyCH4IV (New England Biolabs, Whitby, ON, Canada). Digestions were performed at 37°C for 4 h in a total volume of 10 *μ*L using 2 *μ*L of PCR products, 1 × reaction buffer, and 3 U of restriction enzyme. Fragments were separated using 1.5% standard 1 × TAE agarose gels.

#### Classification

All trees, seedlings, and seeds were screened with the diagnostic SNP array, and all individuals (*n* = 218) with alleles for poplar species not native to North America (*P. nigra*, *P. maximowiczii*, or *P. laurifolia*) were excluded from further analyses. We address these data in a companion paper on exotic poplar hybridization (Roe et al. 2014). The remaining individuals bearing only alleles for native poplar species were assigned to one of three genotype classes: pure *P. balsamifera* (B), pure *P. deltoides* (D), and native hybrid (D × B). Native hybrids had both *P. balsamifera* and *P. deltoides* alleles. The diagnostic markers were fixed, so we were able to manually assign individuals to these categories by inspecting for alleles specific to each species. Paternal contributions to seed genotypes were determined using the known maternal genotype and haplotype subtraction (Meirmans et al. 2010). These manual assignments were conducted by two independent observers (M Lamothe and AD Roe). To complement our manual assignments, we used two Bayesian clustering algorithms [Structure version 2.3.3, (Pritchard et al. 2000); New Hybrids version 1.1 beta, (Anderson and Thompson 2002)] to assign individuals to genetic clusters and quantify admixture in each individual (Fig. S1).

### Phenology

Flowering phenology was examined to estimate the overlap between the flowering periods of each genotype class (B, D, D × B) and between males and females within each class. Phenology stages were determined as in Gom and Rood (1999), with minor modifications. In 2011, 13 females (B = 4, D = 4, D × B = 5) and eight males (B = 4, D = 3, D × B = 1) were observed with binoculars every 2–5 days from April 29 to May 20 and scored for their flowering stage. Flowering stages were recorded as follows: bud dormancy (stage 0), bud break (stage 1), bud expansion, or elongation of the catkin (stage 2), pollen dehiscence (male)/receptivity (female) (stage 3), senescence (male)/capsule ripening (female) (stage 4). An individual was scored for a given stage when more than 50% of the reproductive structures had reached that stage.

### Hybridization rate

Hybridization was quantified using the spontaneous hybridization rate. The spontaneous hybridization rate is the “classical” method of estimating the frequency of hybrids and was obtained by dividing the number of hybrid offspring by the total number of offspring examined.

### Reproductive fitness

#### Biomass and yield

Biomass and reproductive output were measured on catkins collected from trees in each genotype class (i.e., B, D, D × B) in 2009 and 2011 (Table 1). Prior to dehiscence, we randomly sampled up to 10 catkins from each tree and placed them in individual sealed paper bags. The number of capsules per catkin was recorded, and then, the catkins were allowed to air-dry under ambient laboratory conditions for 10–40 days at 21°C until the capsules opened.

**Table 1:** Number of trees measured for reproductive biomass, reproductive yield, seed germination, and fungal disease susceptibility. Fungal susceptibility was measured for three *Melampsora* species*: M. larici-populina* (*Mlp*), *M. medusae* f.sp. *deltoidae* (*Mmd*), and *M. occidentalis* (*Mo*). Trees were grouped into three genotype classes: pure *Populus balsamifera* (B), pure *P. deltoides* (D), and native hybrids (D × B). Numbers in brackets indicate the number of trees sampled more than once

	2009			2010			2011		
			
	B	D × B	D	B	D × B	D	B	D × B	D
Biomass	2	3	3	–	–	–	5 (1)	5 (2)	3 (1)
Yield	2	3	3	–	–	–	5 (1)	5 (2)	3 (1)
Germination	3	7	17	4 (3)	5 (4)	13 (11)	5 (4)	3 (3)	4 (3)
Disease susceptibility									
*Mlp*	3	8	7	–	–	–	–	–	–
*Mmd*	3	8	7	–	–	–	–	–	–
*Mo*	5	8	7	–	–	–	–	–	–

Following drying, each catkin was separated into four components: cotton, capsules, stem axis, and seed. Each component was placed on separate aluminum drying dishes. Fully dehisced cotton was extracted, and any cotton remaining within the capsules was removed with forceps. All extracted cotton was placed in a blender to separate the seed and cotton. Any seed remaining within the cotton was extracted with forceps. The separated seed was removed from any remaining capsule debris, and the debris was included with the capsule component. The four drying dishes from each catkin were then placed in a forced draft oven set at 70°C for 16 h and then placed in a dessicator and allowed to cool. Once cool, the components of each catkin were weighed, including 100 seeds.

#### Germination

Germination tests were conducted on seeds extracted from catkins that were collected from trees in each genotype class in 2009–2011. As before, we collected catkins from trees (Table 1) prior to natural dehiscence, sealed the catkins in individual paper bags, and allowed them to dehisce in drying cabinets at a temperature of 20–25°C:40% RH. Seeds were visually assessed at the time of collection to ensure that they were mature (i.e., visible cotyledons and radicle with embryo filling the seed). We extracted seeds following methods in Daigle and Simpson (2009) and recorded 1000 seed weight (TSW, in grams) for each seed lot.

Seeds were evaluated for germination success on artificial growing media under controlled conditions. Four replicates of either 50 or 100 seeds were selected with the aid of a vacuum plate and placed on moistened Versa-Pak™ (National Packaging Services Corp., Green Bay, WI) in Petawawa germination boxes (Wang and Ackerman 1983) with four replicates of either 50 or 100 seeds per box. Each germination box was placed in a G30 germinator (Conviron, Winnipeg, MB) (8 h light at 30°C, 16 h darkness at 20°C:85% RH) for 10 days. Each replicate was examined for signs of germination starting on day three and ending on day ten, at which time germination state was recorded for each replicate. Germination was scored as successful (cotyledons had separated, the hypocotyl was upright, and the radicle grew into the Versa-Pak), abnormal [one or more of the following: fused cotyledons, curved hypocotyl, “stump root”, or was leaning or lying on the surface of the germination medium (Simak 1982)], or failed (no sign of germination).

### Disease susceptibility

#### Controlled inoculations with *Melampsora* species

We assessed disease susceptibility of adult trees to fungal diseases using controlled leaf inoculation with mono-uredinal *Melampsora larici-populina* Kleb. (Mlp), *M. medusae* Thuem. f. sp. *deltoidae* (Mmd), and *M. occidentalis* Jacks (Mo) strains from the Laurentian Forestry Centre culture collection. We obtained leaves for testing susceptibility from dormant winter cuttings of 20 poplar trees (Table 1) from the three genotype classes that were clonally propagated in a greenhouse using internal protocols. Four different rooted cuttings from each tree were selected for four inoculation replicates. We obtained fungal inoculates by propagating mono-uredinal strains for Mlp (strain Mlp05BERT3729, Berthierville, QC) and Mmd (Mmd05TRE539, Trécesson, QC) on detached leaves of the Euramerican poplar clone “Robusta” (*P. deltoides* × *P. nigra*). Mo (strain Mo05CA07, Shasta Lake, CA) was propagated on detached leaves of *P. balsamifera* (BPSF-072). Ten days post-inoculation, we removed urediniospores from the leaves with a stainless steel single-edge dissecting needle and used them to prepare a water solution of 5000 urediniospores/mL supplemented with Tween 20 (0.02%). Fully expanded leaves with a leaf plastochron index of (LPI) three were removed from each poplar clone, placed onto wet paper towel in Petri dishes, and then spray-inoculated on their abaxial surface with the spore solution using an airbrush at 20 psi. Inoculation trials were fully randomized. Petri dishes were incubated for 11 days (19°C, 16:8 h L:D, 80% RH) after which time we counted the number of uredia that had formed and measured the leaf area using Assess 2.0 software (APS Press, Saint Paul, MN).

#### Field surveys of fungal incidence

Adult trees were visually surveyed for incidence of fungal damage caused by *Septoria* spp. leaf spots and *Melampsora* spp. leaf rust. Incidence surveys were conducted in mid-September 2009. For *Septoria* spp., we recorded the presence (1) or absence (0) of leaf spot. For *Melampsora* leaf rust, 10 leaves per tree were observed and the total leaf area covered by uredia was placed in one of three damage classes: no uredia (1), <50% leaf area (2), and >50% leaf area (3).

### Statistical analyses

We analyzed the data using a combination of generalized linear models and mixed-effect models (Pinheiro and Bates 2000; Bolker et al. 2009) (Bates et al. 2012) with post hoc comparisons of the treatment levels when we detected significant main effects. All analyses were conducted in the R statistical computing language (R Development Core Team 2012) using functions in the lmer (Bates et al. 2012), multcomp (Hothorn et al. 2008), nlme (Pinheiro et al. 2012), MASS (Venables and Ripley 2002), and stats (R Development Core Team 2012) packages. We addressed issues of non-normality and overdispersion when necessary (McCullagh and Nelder 1983; Elston et al. 2001; Venables and Ripley 2002). See the supplemental material for a summary of our methods (Table S1) and methodological details of the analyses (Data S2). The code for our analyses has been deposited in Dryad (http://doi:10.5061/dryad.63fr7) and is also available (Data S3).

## Results

### Genotyping

We sampled 142 adult trees (82 females, 59 males, and one unknown), 902 seeds, and 404 seedlings. All adult, seed, and seedling samples (*n* = 1448) were genotyped with the diagnostic SNP array. In total, there were 50,680 SNP genotype calls (1448 individuals × 35 SNPs) and 10,494 counts of missing data, giving a success rate of 79.3%. Of these missing SNPs, the success rate for adults (99.0%), seeds (99.7%), and seedlings (26.7%) varied. Most of the 10,494 missing counts were from the seedlings where 10,341 of 14,140 SNP genotypes failed. The discrepancy between the seedlings and the other two groups was attributed to issues with the DNA extraction kit used for the seedlings, rather than problems with the SNP array itself. If we ignore the seedling category, the failure rate for adults and seeds was 0.4%, which is comparable to previous studies (Meirmans et al. 2010; Thompson et al. 2010; Talbot et al. 2012). Individuals with >10 missing SNP loci were removed (*n* = 326 individuals – one adult tree, two seeds, 323 seedlings).

From the sampled trees, three pairs of trees were identified as possible clones and two pairs of trees were identified as half siblings. Two pairs of D trees were identical for all SNP markers in the D intraspecific panel (data not shown). One pair of D × B trees was identical for all markers in both the D and B panels. We removed one of each pair from the data set (*n* = 3). We also identified two pairs of D × B trees as half siblings. These pairs were identical for all SNP markers in the D panel, but were unique for the B panel (i.e., they had the same mother but different fathers). As these trees were not identical clones, we retained them in the data set. The final data set contained 125 adults, 73 seedlings, and 698 seeds, for a total of 896 native individuals.

### Genotype assignment

There was complete agreement between the three assignment methods (manual, two types of Bayesian admixture analyses: Structure and NewHybrids) in classifying the adult trees and seedlings into separate genotype classes. The assignment methods conflicted when assigning seeds to genotype classes. Bayesian admixture analyses tended to erroneously assign hybrid seed produced by backcrossed females as B or D genotypes. We attributed this to sensitivity issues with our threshold assignment method or the Bayesian clustering algorithm. An additional assignment error arose for 11 seeds that were obtained from hybrid mothers, but were classed as pure seed. The SNPs were scored as homozygous so the hybrid nature of the seed was not detected, either due to typing errors or to the random nature of segregation. These conflicting assignments highlight the limitations of detecting advanced-generation hybrids with stringent thresholds and a limited number of markers (Vähä and Primmer 2006).

### Stand characteristics

The majority of native adult trees sampled at BPSF were pure D (78%) with the remainder split evenly between pure B (10%) and D × B (12%). We took a representative sampling of trees from the site, although were limited by our collecting capabilities. Most native hybrids were identified as *F*_1_ individuals, although two individuals were classified as backcrossed into *P. balsamifera* (BC-B) (Table 2). For our analyses, we included these backcrossed individuals with the *F*_1_ hybrids (collectively D × B). Chloroplast haplotyping determined that all 15 adult hybrids at BPSF had D cpDNA genotypes, indicating a D maternal lineage.

**Table 2:** Genotype classification of the final data set from the Base de plein-air de Sainte-Foy based on consensus between manual and Bayesian admixture assignments (Data S1). (A) Total numbers of each genotype class in the adult, seedling, and seed populations. (B) Number of seeds in each genotype class produced by a subset of female trees and the corresponding putative male tree are indicated

	*n*	B	D	*F*_1_	*F*_2_	BC–B	BC–D
*A. Genotype classification*							
Adults ♂	53	5	40	7	–	1	–
Adults ♀	72	8	57	6	–	1	–
Total	125	13	97	13	–	2	–
Seedlings	73	62	11	–	–	–	–
Seeds	698	138	231	3	10	256	60

	Seed							Putative male genotype		
*B. Summary of seed genotypes*										
Female (*n*)	*n*	B	D	*F*_1_	*F*_2_	BC–B	BC–D	B	D	Hybrid^1^
B (3)	139	138	–	–	–	1	–	138	–	1
D (4)	234	–	231	3	–	–	–	3	231	–
*F*_1_ D × B (6)	279	–	–	–	10	209	60	209	60	10
BC-B (1)	46	–	–	–	–	46	–	46	–	–

1 Hybrids may include *F*_1_, *F*_2_, or backcrossed individuals, but these classes could not be differentiated.

### Stand DBH and height

**Figure 2:**
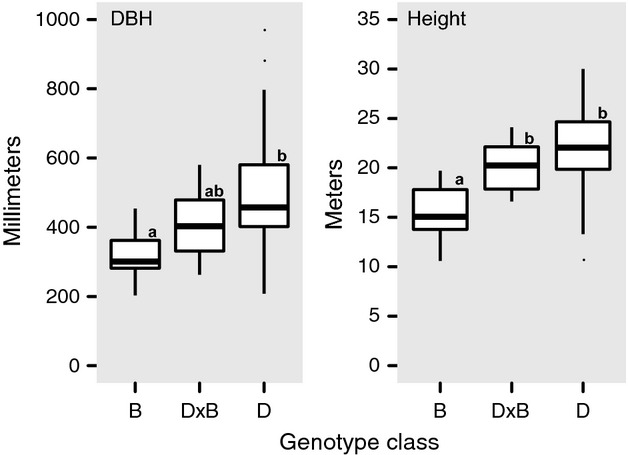
Diameter at breast height (DBH) and height of sampled adult trees. Tree genotype classes are as follows: pure *Populus balsamifera* (B), pure *P. deltoides* (D), and native hybrids (D × B). Post hoc tests (Tukey's Honestly Significant Difference) were used to identify differences between genotype class means when significant differences were detected with a general linear model.

Tree height and DBH were significantly different between genotype classes (Fig. 2, *P* < 0.0001). Pure B trees had a significantly smaller DBH than D trees (310.4 mm ± 74.2 vs. 490.6 mm ± 143.6; HSD *P* < 0.001; all values mean ± 1 SE) and were shorter than D trees (15.4 m ± 2.9 vs. 22.2 m ± 3.9; HSD *P* < 0.001). Pure D trees were variable in both DBH and height, but were generally larger and taller than other trees in the stand, except for native hybrids. Native hybrids were intermediate in DBH (412.6 mm ± 99.3) and height (20.1 m ± 2.4), and were significantly taller than B trees (HSD *P* = 0.002). Age classification mirrored the trends observed in DBH and height data. Pure B were younger trees (<40 years old), as were the majority of the D × B trees (12/14), while over half (51/100 trees) D were old trees (>40 years old) (Table S2).

### Regenerated seedlings

All native seedlings in six transects (one transect had no *Populus* samples) were either B or D, and no D × B were detected (Table 2). Among seedlings, B were more numerous than D, which contrasted with the adult tree population.

### Phenology

**Figure 3:**
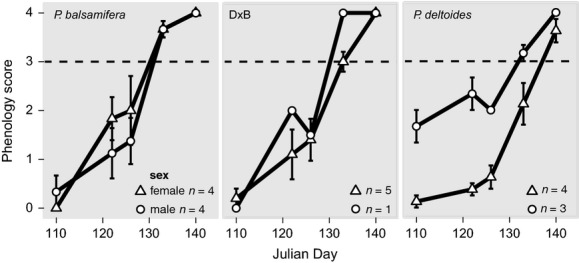
Flowering phenology of male and female *Populus balsamifera*, *P. deltoides*, and native hybrids. Dashed line indicates pollen shedding and female receptivity.

Flowering synchrony showed similar phenological timing and overlap among genotypes and between genders (Fig. 3). Male (*n* = 4) and female (*n* = 4) B were synchronous throughout the period of flowering, with both pollen dehiscence and flower receptivity occurring simultaneously. Male (*n* = 3) and female (*n* = 4) D trees were asynchronous in the flowering stage. Male trees were more temporally advanced than females throughout the flowering period, meaning that pollen dehiscence occurred prior to flower receptivity. Male (*n* = 1) and female (*n* = 5) D × B were moderately synchronous, although male flower phenology was based on a single male tree. By calendar day 130, males and females of B, native hybrids, and male D had reached or exceeded pollen dehiscence/receptivity. Female D trees were the exception; by calendar day 130, the average flowering score for female D was 2.12, while B and native hybrids were fully receptive (3.0 and 3.67), respectively (Fig. 3).

### Spontaneous hybridization rate

The majority of reproductive events were intraspecific crosses (369 seeds, 98.9%), with three *F*_1_ hybrid seeds produced by female D (0.8%), and one hybrid backcross into B (0.2%; Table 2). Among D × B, the majority of reproductive events were BC-B (255 seeds, 78.5%), with fewer BC-D (60 seeds, 18.5%) and *F*_2_ hybrid seeds (10 seeds, 3.1%). The spontaneous natural hybridization rate (the percentage of hybrid seed detected in female trees) was slightly higher for D (1.3.%) than B (0.7%).

### Reproductive fitness

We measured reproductive biomass and yield for 18 trees in 2009 and 2011. The sampling was partially replicated with four trees sampled in both years, four trees sampled only in 2009 and ten trees sampled only in 2011 (Table 1). The analysis of the double-sampled trees showed a correlation between these fitness traits among years (*R* = 0.67), but it was not significant (*t* = 1.29, *df* = 2, *P* = 0.32), and there was a weak but significant effect of “year” for the four trees (Data S2 and S3). As the results of this analysis were somewhat ambiguous, we performed the analysis of biomass and yield twice, once with the full data set treating all samples as independent, and again on a partial data set from which we removed the duplicated samples, so that each tree was only represented once. We present the results from the analysis of the full data set in the figures below; the mixed-effect model summaries for both the full and partial data sets are given in supplemental tables (Tables S3 and S4).

#### Biomass

**Figure 4:**
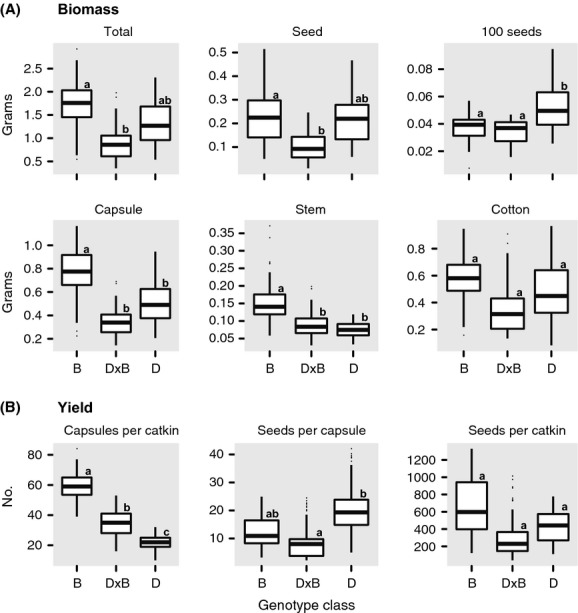
Box plot of adult tree reproductive biomass and yield for pure *Populus balsamifera* (B), pure *P. deltoides* (D), and native hybrids (D × B). Reproductive biomass measures include the following: total biomass, seed biomass, 100-seed biomass, capsule biomass, stem biomass, and cotton biomass. Reproductive yield measures number of capsules per catkin, seeds per capsule, and seeds per catkin. Post hoc tests were used to identify differences between genotype class means when significant differences were detected with linear mixed-effect models (Tables S3 and S4).

We measured six biomass traits: total catkin biomass, total seed biomass, 100 seed biomass, capsule biomass, stem biomass, and cotton biomass. All analyses showed a significant effect of either genotype class or year on these reproductive traits (Table S3). Compared with *D*, catkins from *B* had lighter seed (100 seed biomass, *P* = 0.035) and greater capsule (*P* = 0.01) and stem biomass (*P* < 0.001) (Fig. 4A). Catkins from D × B differed from at least one parent, although if this difference was significant depended on which data set we analyzed (Tables S3 and S4). Compared with *B*, D × B had lower total biomass (full, *P* = 0.001; partial, *P* = ns), capsule biomass (full, *P* < 0.001; partial, *P* = 0.001), total seed biomass (full, *P* = 0.016; partial, *P* = ns), and stem biomass (full, *P* = 0.003; partial, *P* = ns) (Fig. 4A). Native hybrids had significantly lighter seed (100 seed weight, *P* < 0.01) than D (Fig. 4A). In the full data set, genotype class had a significant effect on total biomass, capsule biomass, 100 seed biomass, and stem biomass; year had a significant effect on cotton biomass. The same results were seen in the partial data analysis for capsule biomass, 100 seed biomass, and stem biomass, but we detected no effect of either genotype class or year in this data set for total biomass, cotton biomass, and seed biomass (Table S3).

#### Yield

We measured three yield traits: number of capsules per catkin, number of seeds per capsule, and total number of seeds per catkin. The number of capsules per catkin differed for each genotype class, with B having more than D (full, *P* < 0.001; partial, *P* < 0.001), and hybrids being intermediate between B (full, *P* < 0.001; partial, *P* < 0.01) and D (full, *P* < 0.001; partial, *P* < 0.001) (Fig. 4B). Native hybrids had fewer seeds per capsule than B (Fig 4B) although this was not significant for the full or partial data sets. Native hybrids had fewer seeds than D (full, *P* < 0.001; partial, *P* < 0.01). The factors influencing yield were not consistent among the three measures. Overall, the total number of seeds per catkin was not significantly different between the genotype classes, although differences did exist between classes for the number of capsules per catkin and seeds per capsule. Year had no effect in any of the analyses (Table S4).

#### Germination

**Figure 5:**
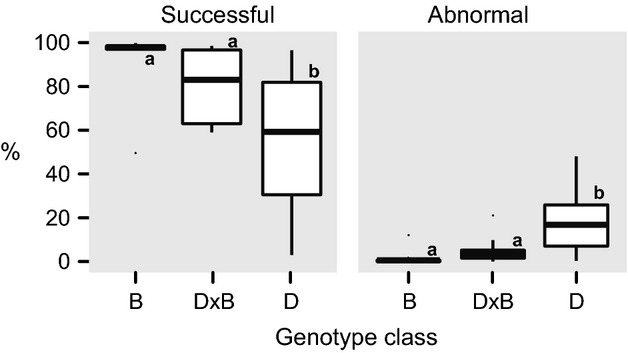
Box plot of adult tree seed viability for pure *Populus balsamifera* (B), pure *P. deltoides* (D), and native hybrids (D × B). Seed viability was assessed based on percent germination and percent abnormal germinants. Post hoc tests were used to identify differences between genotype class means when significant differences were detected with general linear models (Table S5).

Germination traits consisted of two measurements: germination success and number of abnormal germinants. We quantified seed germination for seed collected in 2009, 2010, and 2011 from a total of 41 trees (Table 1). B had better germination and fewer abnormal germinants than D (Fig. 5). Compared with B, D × B had more abnormal germinants than B in 2009, but this was not observed in 2009–2011. Compared with D, D × B had better germination and fewer abnormal germinants in both data sets. Tree genotype was the only factor with an effect on the germination of seed (Table S5). The distribution of sampling among years and genotype class was not equal (Table 1). Also, some trees were sampled in multiple years. Despite the repeated sampling, the data were too sparse to allow us to test the effect of both year and genotype class in the same analysis. Therefore, we performed two separate analyses. First, we tested the effect of genotype class and TSW on germination using just the seed collected in 2009. Then, we analyzed the effect of genotype class, TSW, and year by excluding some of the data to produce a more balanced data set. To create a balanced data set, (1) we selected all data from trees that were sampled once in either 2009 or 2010; (2) we selected all records from trees sampled in 2011; (3) from the remaining trees not selected in the previous steps, we then randomly selected one record from each tree. We included all the records from 2011 because the fewest samples were taken that year. The selection of records in step (4) was partially supervised in that the random selection was performed in R, but repeated until the number of samples in each year–genotype class combination was approximately balanced (Data S3).

#### Disease susceptibility

**Figure 6:**
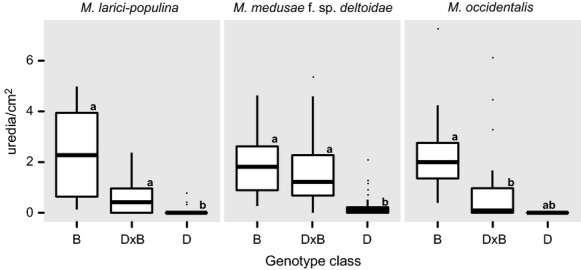
Box plot of adult tree fungal disease susceptibility for pure *Populus balsamifera* (B), pure *P. deltoides* (D), and native hybrids (D × B). Disease susceptibility was assessed based on the number of uredia/cm^2^ for using controlled inoculations of three *Melampsora* species. Post hoc tests were used to identify differences between genotype class means when significant differences were detected using linear mixed-effect models (Table S6).

A total of 18 trees were inoculated for Mlp, 18 trees for Mmd, 20 trees for Mo. In the inoculation experiments, genotype class had a significant effect on the number of uredia/cm^2^ in all three experiments (Fig. 6). More uredia grew on B and D × B than D for all three fungal inoculations, but we only observed significant differences for Mlp and Mmd (Fig. 6). Significant differences between B and D × B were detected for Mo, although not for D and the other two genotype classes. D may be less susceptible to this fungal species than either B or D × B, although these results should be interpreted with caution (Table S6). For all inoculations, the model containing genotype class was a better fit than a null model containing only the random effects (Table S6). Field surveys of *Melampsora* spp. incidence showed patterns similar to those observed in the controlled inoculations (Table S7). Pure B and D × B showed similar levels of disease incidence, and pure D had lower levels of infection. *Septoria* spp. leaf spot was observed on nearly every tree, with the exception of four D trees.

## Discussion

Introgression of interspecific genetic material is largely determined by the initial formation of viable *F*_1_ hybrid individuals, followed by their establishment and reproduction in native habitats. By screening historic (adult) and contemporary (seed and seedling) natural poplar populations, we quantified the hybridization and introgression dynamics in a native poplar hybrid zone. Both pure parents and native hybrids acted as fertile reproductive partners producing pure and admixed offspring. Asymmetrical hybridization was observed in our system, although the direction of the asymmetry was dependent on the cross. The formation of *F*_1_ hybrids was biased toward *P. deltoides*, while subsequent introgression was biased toward *P. balsamifera*. Postzygotic fitness differed between each genotype class and was likely impacted by both intrinsic and extrinsic factors. Native hybrids were not universally unfit, but were intermediate in fitness relative to pure trees based on our fitness measures. However, native hybrids were absent in the seedling population, suggesting that hybrid seedlings were experiencing additional selective forces that limited recruitment and lifetime fitness of hybrids in this system. Our results suggest that complex interactions among several reproductive barriers maintain this hybrid zone.

### Hybrid formation

Poplars are well known for their ability to hybridize with related species, although it is generally restricted to members of the same section. The exception is for crosses between species in sections Tacamahaca and Aigeiros (Eckenwalder 1984). At BPSF, both native *F*_1_ hybrids and advanced-generation backcrosses were detected in adult and seed populations. While all genotype classes produced seed, we observed asymmetry in hybrid formation and subsequent introgression (Table 2). Female *P. balsamifera* did not form *F*_1_ hybrids, but males were the primary pollen donor for hybrid backcrosses (Table 2). Conversely*, P. deltoides* were capable of forming *F*_1_ hybrids (Table 2 and cpDNA), but were infrequent pollen donors in backcrosses. Detailed analyses of intersectional hybrid zones have demonstrated that asymmetrical crossing barriers exist between Tacamahaca and Aigeiros species (e.g., Keim et al. 1989; Floate 2004; Thompson et al. 2010). For example, *F*_1_ crosses will form if a member of Aigeiros is the seed parent, but reciprocal *F*_1_ crosses are rare or absent if Tacamahaca is the seed parent (Zsuffa et al. 1999). Asymmetrical introgression has also been observed among Aigeiros and Tacamahaca crosses, where gene flow was unidirectional toward the Tacamahaca parent and with little or no gene flow occurring in the opposite direction (Keim et al. 1989; Floate 2004).

Causes of asymmetric hybridization and reproductive isolation may involve complex interactions between prezygotic and postzygotic barriers (Rieseberg and Carney 1998; Coyne and Orr 2004). Prezygotic barriers include flower morphology, relative population size, and phenology that act before mating occurs (Rieseberg and Willis 2007). Other prezygotic barriers known to impact hybrid formation in poplars include pollen competition, prefertilization incompatibilities, and pollen–pistil interactions (Stettler et al. 1996b; Vanden Broeck et al. 2005). Previous work on crossing barriers in poplars has shown that these mechanisms do not constitute strong barriers to hybridization, particularly among Aigeiros and Tacamahaca crosses (Knox et al. 1972; Guries and Stettler 1976; Rajora 1989; Vanden Broeck et al. 2003), although these mechanisms have not been explicitly tested in the *P. balsamifera* and *P. deltoides* hybrid zone.

Relative species abundance has been shown to impact the rate and directionality of hybridization and subsequent introgression, leading to asymmetrical gene flow among wind-pollinated trees (Lepais et al. 2009; Meirmans et al. 2010; Field et al. 2011; LeBoldus et al. 2013). At BPSF, *P. deltoides* was the dominant adult tree species, so we had expected to observe demographic swamping of *P. balsamifera* given differences in relative population sizes (Hubbs 1955; Levin et al. 1996). If demographic swamping occurred, then we should have observed more *F*_1_ hybrids in female *P. balsamifera* and more hybrids backcrossing with *P. deltoides*. In fact, we observed the opposite; *F*_1_ hybrids formed only with female *P. deltoides,* and the majority of hybrids backcrossed with *P. balsamifera* (Table 2 and cpDNA data). Although the majority of trees at BPSF were *P. deltoides*, this species is rare in the surrounding landscape. The BPSF is at the northern edge of the distribution of *P. deltoides* (Little 1971; Rousseau 1974). By comparison, there were stands of *P. balsamifera* within 5 km of BPSF. Nearby stands of *P. balsamifera* could have contributed to the available pollen cloud at BPSF given that pollen from wind dispersed trees can travel significant distances (Slavov et al. 2009; Talbot et al. 2012). The result would be that the composition of the pollen cloud at the BPSF was more representative of the relative species abundance in the surrounding landscape than of the local stand. Consequently, the BPSF *P. deltoides* population could be pollinated by external *P. balsamifera* pollen sources.

Relative abundance of pollen is dependent on the timing of pollen availability, which is in turn controlled by phenology. Plant phenology is also a well-known premating barrier (Rieseberg and Carney 1998; Vanden Broeck et al. 2003; Hall and Willis 2006). Timing of reproduction was not likely a major reproductive barrier at BPSF as pure species and native hybrids exhibited phenological overlap (Fig. 3), which was consistent with previous observations of poplars (Braatne et al. 1996; Gom and Rood 1999; Talbot et al. 2012). We observed one exception, which was the timing of receptivity in female *P. deltoides*. Male *P. deltoides* were asynchronous with *P. deltoides* females such that pollen was available before female flowers were receptive (Fig. 3). As we noted earlier, the *P. deltoides* at BPSF are at the northern edge of their range which may influence the timing of reproduction (Chuine 2010, see below). If conspecific pollen was not available when female flowers were receptive, then there would have been opportunities for interspecific hybridization to occur, particularly if outlying *P. balsamifera* stands were more synchronous and contributed to the available pollen cloud. We are currently developing dense SNP marker panels that would allow us to address whether the hybrids at BPSF were pollinated by local individuals or by outlying individuals via long distance pollen dispersal.

Reproductive barriers that act early in an organism's lifecycle contribute more to reproductive isolation than later barriers (Coyne and Orr 2004). For example, genetic incompatibilities that prevent the formation of hybrids or lead to infertile or inviable offspring are strong reproductive barriers. Genetic incompatibilities may disproportionately affect specific crosses, leading to asymmetric hybrid formation and introgression (Tiffin et al. 2001). At BPSF, all *F*_1_ hybrids had *P. deltoides* as the female parent (Table 2 and cpDNA). This is consistent with observations in natural stands within hybrid zones and controlled crosses between *P. deltoides* and other Tacamahaca species (Zsuffa et al. 1999; Riemenschneider et al. 2001; LeBoldus et al. 2013). While reciprocal crosses with a Tacamahaca seed parent can be produced in controlled crosses (Zsuffa et al. 1999; Riemenschneider et al. 2001) and have been detected in natural stands (Thompson et al. 2010), they are less frequent and more difficult to form than interspecific crosses with Aigeiros as the seed parent (Zsuffa et al. 1999). It is hypothesized that postzygotic barriers to reproduction likely contribute to asymmetrical hybrid formation in this and other poplar hybrid zones (Stettler et al. 1996b; Zsuffa et al. 1999), although the mechanisms creating these barriers have been largely unexplored. One potential barrier to hybrid formation is conflicting developmental schedules in the maternal tissue and seed embryo (Stettler et al. 1996b). Timing of catkin, capsule, and nonpersistent endosperm development is a process governed by the maternal genome, while hybrid embryo development is controlled by the admixed genome in the developing seed. If the hybrid seed's parental species have conflicting developmental schedules, then timing conflicts could arise. For example, if the maternal tissues mature before the embryo is ready, then embryo maturation could be disrupted or the embryo aborted (Stettler et al. 1996b). This mechanism could explain the asymmetry observed among *F*_1_ crosses at BPSF and has been hypothesized to explain the asymmetry in hybrid crosses between *P. deltoides* and *P. trichocarpa* (Riemenschneider et al. 2001). At BPSF, *P. balsamifera* matures and dehisces sooner than *P. deltoides* (Fig. 3), so female *P. balsamifera* with capsules containing hybrid seed may have dehisced before hybrid seeds were fully mature. Conversely, if the hybrid embryo matures faster than the maternal tissues, then the reverse cross may not suffer the same fate as the embryo would be fully developed prior to capsule dehiscence, although there may not be sufficient maternally derived endosperm to support the early development of the embryo leading to delayed or abnormal embryos. This hypothesis is complicated by the fact that *P. deltoides* and *P. balsamifera* also exhibit latitudinal variation in phenology of flowering (Braatne et al. 1996; Soolanayakanahally et al. 2013). For instance, Riemenschneider et al. (2001) showed that male *P. trichocarpa* x female *P. deltoides* crosses with fathers from northern provenances had higher rates of success than those from crosses with fathers from southern provenances. Their finding lends support to our hypothesis regarding the formation of hybrids at BPSF. Controlled crosses between intra- and interspecific parents showing variation in spring phenology would be needed to fully evaluate this reproductive barrier.

Organisms with heteromorphic sex chromosomes may experience preferential loss or reduction in fitness in the heteromorphic sex in the *F*_1_ generation, a phenomenon termed Haldane's Rule (Haldane 1922). Brothers and Delph (2010) recently demonstrated that three species of dioecious plants with heteromorphic sex chromosomes conformed to Haldane's Rule. They suggested that this model could apply to other plants with sex chromosomes, such as poplar. This is relevant because sex linkage has been proposed as another cause of asymmetrical hybrid formation in poplars (Thompson et al. 2010; Macaya-Sanz et al. 2011). Poplars are strictly dioecious, and sex is genetically controlled, although the mechanism of sex determination in poplar is still unknown. However, recent work suggests that poplars have an incipient sex chromosome located on chromosome XIX, although the location of the putative sex determination region varies between sections (Tuskan et al. 2012). For these reasons, identification of the heterogametic sex has not been straightforward; some evidence suggests that females are heterogametic (Yin et al. 2008) or that both forms exist within the genus (i.e., ZW or XY depending on the species, Pakull et al. 2011). Regardless, assuming poplars have a heterogametic sex system and Haldane's Rule applies to this system, we would predict a sex-linked bias in the viability or fertility of *F*_1_ hybrids. At BPSF, we observed no distortion of the sex ratio among *F*_1_ hybrids (sex ratio was 50:50) and both hybrid males and females acted as fully fertile reproductive partners (Table 2). This would suggest that poplar does not conform to Haldane's Rule. That said, sterility has been observed in a number of ♀*P. balsamifera* × ♂*P. deltoides* cultivars produced from controlled crosses (Eckenwalder 2001), so it is possible that fitness reductions in a particular sex, rather than complete inviability, occur. As such, it would be worthwhile to continue exploring the impact of sex linkage on poplar hybridization and introgression.

### Hybrid fitness

After hybrids have been formed, the evolutionary consequences of hybridization depend on hybrid fitness (Arnold and Martin 2010). Hybrid fitness is controlled by both intrinsic and extrinsic factors, although the relative importance of each to hybrid zone dynamics is contentious and system dependent. Given the nature of our study site, we were unable to examine extrinsic factors impacting hybrid fitness. Instead, we focused on intrinsic aspects of hybrid fitness traits and sought to identify traits that could explain the observed hybrid zone dynamics at BPSF. We examined four fitness traits for *P. balsamifera*, *P. deltoides*, and native hybrids: reproductive biomass and yield (Fig. 4), seed germination (Fig. 5), and fungal disease susceptibility (Fig. 6). By measuring reproductive biomass and yield, we sought to quantify maternal investment in reproduction. There were significant differences among each class of tree, although due to our low sample size, it is difficult to evaluate the degree of variation in these traits. Pure species differed in their reproductive biomass in most maternal tissues (catkins, capsule, stem, and cotton), although this was only statistically significant for the capsules. Despite higher investment in maternal reproductive tissue, the seed biomass and 100 seed weight were not significantly different between the two pure species. Relative to the pure species, native hybrids had lower biomass and yield than both *P. balsamifera* and *P. deltoides* (Fig. 4), and a number of measures were significantly different in *P. balsamifera* (total biomass, seed biomass, capsule biomass, stem biomass) and *P. deltoides* (100 seed weight, seeds per capsule) (Tables S3 and S4). Hybrid fitness traits showed an overall reduction relative to the two pure species, although none of these reproductive traits were significantly lower than both parents simultaneously, suggesting that for reproductive output, hybrids were not universally unfit at least in terms of their reproductive yield.

In poplars rapid, successful germination is essential to accessing adequate light, moisture, and mineral soil (Braatne et al. 1996). We observed the highest rate of germination in *P. balsamifera* (96% 11/12 trees) (Fig. 5). Native hybrids had a similar germination to *P. balsamifera*, although with our low sample size, it is difficult to evaluate the variability of this trait within the population. By contrast, germination in *P. deltoides* was unexpectedly low, with high numbers of abnormal germinants (Fig. 5). We had predicted better germination success based on high germination success observed in southern *P. deltoides* populations (i.e., >90% success, Farmer and Bonner 1967; Hardin 1984; pers. comm. B. Stanton, B. McMahon). Our unexpected germination results could be attributed to unfit hybrid genotypes, but as ungerminated or abnormal seeds were not genotyped, we can only speculate whether seed germination was directly linked to levels of admixture. Even so it is unlikely that the poor levels of germination in the *P. deltoides* seed could be fully attributed to the effects of hybridization. The hybridization rate in *P. deltoides* was much less (1%) than the germination failure rate (44%) or percent of abnormal seed (30%), so it is likely that other factors influenced germination of *P. deltoides*.

Populations that inhabit regions at the limit of their range may experience decreased fitness (Purves 2009; but see Sexton et al. 2009), similar to what we observed in *P. deltoides* at BPSF. In plants, temperature is a key factor influencing tree reproductive fitness (Morin et al. 2007, 2008; Chuine 2010). Morin et al. (2007) used a process-based model to demonstrate that the northern limits of *P. deltoides* and sixteen other tree species were correlated with spring temperatures. They hypothesized that the inability to complete flowering or full fruit development should limit northern spread of these species. Our data support this hypothesis. Ovule ripening and seed maturation are temperature dependent in *P. deltoides,* and insufficient degree days would affect fruit development (Braatne et al. 1996). The impact of temperature on *P. deltoides* may also explain the phenological mismatch between male and female trees (Fig. 3). Another consequence of being on the northern limit of its range is the small *P. deltoides* population size. Small population size is linked to declines in seed production, germination, and survival (Hensen and Wesche 2006; Field et al. 2008) and attributed to a number of factors including habitat quality, climatic variability, reduced genetic diversity, high mutation load, and inbreeding depression (Ellstrand and Elam 1993; Keller and Waller 2002). When we sampled our stands *P. deltoides* comprised ∼70% of the trees at BPSF, however, the initial colonizers of BPSF may have been just a few related individuals leading to inbreeding within the stand. Inbreeding can lead to reduced fitness, although subsequent outbreeding can restore fitness among populations experiencing inbreeding depression (Seltmann et al. 2009). If the BPSF population is inbred, then a reduction in fitness may occur. This coupled with the fact that the population is at the edge of its range could result in an overall low offspring fitness. We are currently comparing the intraspecific variability observed within *P. deltoides* at BPSF and among the surrounding populations to assess relatedness and fitness of trees in this northern population.

The primary measure of a plant's fitness is its ability to produce viable seed. However, almost of equal importance is the ability of its seeds to establish in a given environment and compete for limited resources. At the BPSF, the composition of the seedling population was opposite to that of the adult population (Table 2). Given the low rate of germination and the high numbers of abnormal seed in *P. deltoides*, it was not surprising it contributed a disproportionately small fraction of the seedling population. The high number of *P. balsamifera* suggested a greater competitive advantage over *P. deltoides* in seedling establishment. This disparity may provide *P. balsamifera* a means to outcompete *P. deltoides* or grow in conditions that are unsuitable for *P. deltoides*. For example, seedling survival may be tied to successional stage. If *P. deltoides* was less tolerant to shade than *P. balsamifera,* then we would expect to see progressively fewer *P. deltoides* seedlings as shaded conditions increased. Conversely, if *P. balsamifera* seedlings were able to establish more frequently in the more shady conditions, then we would see more younger *P. balsamifera* seedlings. At BPSF we observed that *P. deltoides* trees were larger and older than both *P. balsamifera* and native hybrids. This supports the idea of early colonization by *P. deltoides* followed by later colonization of *P. balsamifera* and hybrids. We would be interested in testing this hypothesis in a controlled setting to assess the shade tolerance of seedlings from each genotype class.

We were surprised by the lack of native hybrid seedlings in our plots, given the formation of viable hybrid seed (Fig. 5). Comparing the numbers of hybrids in the seed population versus the seedling population would suggest that we have overestimated the hybridization rate for BPSF. Frequently, estimates of hybridization rates are estimated at the seed life stage, but these estimates are often not reflected in later generations, due to selection against hybrids (Curtu et al. 2009). We demonstrated that hybrids germinated as well as or better than the pure species, so the lack of hybrids in the seedling population suggests that there are other factors controlling establishment. If hybrid seedlings were less competitive or were more susceptible to disease, then their ability to establish and survive would be hindered. Poplar hybrids can be susceptible to fungal diseases and herbivores (Whitham 1989; Kalischuk et al. 1997; Gom and Rood 1999; Feau et al. 2010). Adult hybrids showed intermediate susceptibility to *Melampsora* infection, relative to *P. deltoides* and *P. balsamifera* (Fig. 6), similar to observations for *Septoria* canker on pure species and *P. deltoides* × *P. balsamifera* Sarg. cv. Northwest (Leboldus et al. 2009 LeBoldus et al. 2013). *Melampsora* leaf rust is known to kill young seedlings, so if young hybrid seedlings were more susceptible than seedlings of pure species, then they could have been removed from the stand in the first few years of growth (Newcombe et al. 1994, 2001). Susceptibility to fungal diseases may also explain the population differences between *P. balsamifera* and *P. deltoides* in the adult and seedling populations. Under controlled conditions, *P. balsamifera* showed susceptibility to all three *Melampsora* spp., while *P. deltoides* shows little to no susceptibility to rust infection. Interplay between different selective forces may be maintaining the population structure of poplars at BPSF. Poor germination and establishment in *P. deltoides* relative to *P. balsamifera* may be counterbalanced by fungal disease resistance in later life stages allowing persistence of *P. deltoides* within the stand.

## Conclusions

To understand the factors maintaining a stable hybrid zone, it is imperative to quantify the intrinsic and extrinsic barriers to the survival and establishment of pure and hybrid individuals. Despite the extensive work on poplars, few studies document multiple interacting reproductive barriers and selective forces maintaining poplar hybrid zones in a natural environment. BPSF provided a unique opportunity to examine a natural hybrid stand with a known colonization history, providing a temporal aspect to our study not normally achieved in similar studies. Our results suggest that there is interplay between different selective forces that maintains the hybrid zone structure at BPSF. Both prezygotic and postzygotic barriers, such as demographic swamping, phenology, and genetic incompatibilities, impact the formation of native hybrids, although we were unable to clarify the relative contributions of each of these barriers. Despite these barriers, native hybrids did form and were not universally unfit (Schweitzer et al. 2002; Arnold and Martin 2010). A proportion of the hybrid population reached reproductive maturity and produced viable hybrid offspring. We demonstrated that the realized rate of hybridization in the seedling population was much less than a rate based on hybrid seed. Therefore, using hybrid seeds to estimate hybridization rates may overestimate the true number of hybrid individuals in the population. This discrepancy in hybridization rates suggests that additional selective forces affect hybrid success and establishment. We also identified postzygotic fitness factors impacting *P. deltoides* and *P. balsamifera*, such as disease susceptibility and reproductive fitness. These selective forces also contribute to the dynamics of the hybrid zone by shaping the establishment and distribution of the pure species within this system. The only way to tease apart the relative contributions of each selective barrier to overall hybrid dynamics would be to examine controlled crosses in a common garden environment (Whitham et al. 2006; Martin et al. 2007), but this would be a long and expensive experiment given the delayed onset of reproductive maturity and the long-lived nature of poplars. Instead, by examining the dynamics of hybridization within a natural experiment such as the BPSF, we can explore the effects of changes in the system as might occur due to abiotic or biotic perturbations, such as changes in climate or invasion by exotic species (Roe et al. 2014).

## Supporting Information

**Data S1.** Genotype classification using Bayesian admixture analyses.

**Data S2.** Details for linear mixed effect and general linear models.

**Data S3.** R code for fitness trait analyses.

**Table S1.** Summary of statistical methods used in analysis of poplar data. Fixed and random effects are given for the global model after testing for normality and overdispersion. There were three levels of the genotype class fixed effect and two levels of the year fixed effect; 1000 seed weight was a continuous variable that was centered on the mean value before fitting the model. Offset terms, when present, were log transformed within the model. Abbreviations of fungal species as follows: Mlp: *Melampsora larici-populina*, Mmd: *M. medusae* f. sp. *deltoidae*, and Mo: *M. occidentalis*.

**Table S2.** Distribution of age classes estimated for reproductively mature *P. balsamifera* (B), *P. deltoides* (D), and native hybrids (D × B) at BPSF.

**Table S3.** Summary of the linear mixed effects model analysis for the effect of year and genotype on six biomass measurements from catkins of *P. balsamifera* (B), *P. deltoides* (D), and native hybrids (D × B). Tukey's Honestly Significant Difference test was used to identify differences among genotype class means when a significant effect was detected. A. Full data set; B. Partial data set is restricted to single measurements per tree.

**Table S4.** Summary of the linear mixed effects model analysis for the effect of year and genotype on reproductive yield of *P. balsamifera* (B), *P. deltoides* (D), and native hybrids (D × B). Tukey's Honestly Significant Difference test was used to identify differences among tree class means when a significant effect was detected. A. Full data set; B. Partial data set is restricted to single measurements per tree.

**Table S5.** Summary of the general linear model analysis for the effect of genotype and 1000 seed weight (TSW) on seed germination for seeds from *P. balsamifera* (B), pure *P. deltoides* (D), and native hybrids (D × B). Tukey's Honestly Significant Difference test was used to identify differences among genotype class means when a significant effect was detected. In 2009 we tested TSW and genotype class while in 2009–2011 we excluded multiple sampled trees to create a balanced data set in order to test the additional effect of year (see Data S2 for detailed description of rationale).

**Table S6.** Summary of the linear mixed effects model analysis for the effect of genotype on infection severity (uredia) of poplar rust on *P. balsamifera* (B), *P. deltoides* (D), and native hybrids (D × B). Results are based on controlled inoculations of *Melampsora larici-populina* (*Mlp*), *M. medusae* f. sp. *deltoidae* (*Mmd*), and *M. occidentalis* (*Mo*). Tukey's Honestly Significant Difference test was used to identify differences among tree genotype means when a significant fixed effect was detected.

**Table S7.** Field surveys of fungal disease at Base de plein-air de Sainte-Foy. *Melampsora* spp. incidence was recorded as one of three damage classes: absent, <50% leaf area, and >50% leaf area. *Septoria* spp. leaf spot was recorded as presence or absence. Proportion of observed trees in each class is shown in brackets.

**Figure S1.** Schematic demonstrating the thresholds used to assign individuals to one of three classes: pure *P. balsamifera* (B), pure *P. deltoides* (D), and native hybrids (D – B). Pure reference populations were used to develop threshold levels to assign individuals from BPSF. Individuals above the upper threshold were considered pure. If Q-values fell below the lower threshold, then alleles for that species were absent. Genotype classes were based on Q-values and CI intervals relative to the 0.5 threshold (i.e., *F*_1_) and the lower threshold (backcrosses).
