# Supplementary material for: Fitness dynamics within a poplar hybrid zone: I. Prezygotic and postzygotic barriers impacting a native poplar hybrid stand
Source: Ecol Evol. 2014 Apr 3;4(9):1629–47. doi: 10.1002/ece3.1029 (PMC4063464; doi:10.1002/ece3.1029)
Supplement: Supplementary file 1 [file ece30004-1629-SD1.doc]

**Data S1: Genotype classification using Bayesian admixture analyses**

To complement the manual assignment, we used a Bayesian clustering algorithm to assign individuals into genetic clusters and quantify levels of admixture in each individual. We performed this analysis in Structure version 2.3.3 (Pritchar*d et a*l. 2000) and included an additional 160 pure reference individuals (120 *P. balsamifera* and 40 *P. deltoides*) to act as an *a priori* ‘learning population’ to guide admixture quantification (Pritchar*d et a*l. 2000). All individuals (BPSF trees, seedlings, seeds, and reference populations) were analysed simultaneously with an admixture model, Dirichlet parameter (α) was inferred, and the allele frequency prior (λ) was set to 1.0 (default). BPSF samples were labelled POPFLAG=0 and the reference populations were POPFLAG=1. POPINFO was 0 for all BPSF samples, and the reference species (*P. balsamifera* and *P. deltoides*) were labelled 1 and 2, respectively. PFROMPOPFLAGONLY prior was used so the estimated allele frequencies in the BPSF data were based only on the allele frequencies of the reference populations. STARTATPOPINFO prior used the reference populations as a starting point for population origin. Credible intervals (CI) for admixture (Q-values) were estimated. Exploratory analyses of the data set were performed using different values of *K* (i.e. distinct genetic clusters), and determined that *K*=2 was optimal. After setting *K*=2, we conducted 10 Markov chain Monte Carlo (MCMC) runs to check for stationarity in our estimates of admixture. Each MCMC run had an initial burn-in of 50 000, followed by 500 000 replicates.

Assignment to one of the three genotype classes (B, D, DxB) used Q-values from reference populations to set the upper and lower threshold limits for classifying pure and hybrid individuals (Floate *et al*. in prep). In the Bayesian clustering analysis each reference population (*P. balsamifera* and *P. deltoides*) was assigned to a separate cluster, characterized by high Q-values for their individual cluster and low Q-values for the other cluster. We used these Q-values to set the upper and lower thresholds for genotype class assignment (B, D, DxB, Suppl. File 1) and further classified DxB individuals into different hybrid classes (i.e. F_1_ hybrids, backcrosses) based on Q-values and CI relative to the upper, lower and 0.5 (50% admixture) thresholds (Suppl. Figure 1). We confirmed these hybrid classifications by calculating posterior probabilities of assignment to one of 6 genotype classes: pure B, pure D, F_1_, F_2_, backcross to B (BC-B), and backcross to D (BC-D) with New Hybrids version 1.1 beta (Anderson & Thompson 2002). Jeffreys priors were used with a burn-in of 50 000 sweeps followed by 500 000 sweeps. Reference individuals were assigned to known genotype classes (option z), but were not used to estimate mixing proportions and were considered separate from the main BPSF population (option s).

**Data S2: Details for linear mixed effect and general linear models**

The sampling for reproductive biomass, and reproductive yield was partially replicated among the subjects (trees), with six trees sampled in both years (Table 1). To determine if we could treat the 2009 and 2011 samples from the same trees as independent we tested the correlation (Pearson’s R) between total biomass in 2009 and total biomass in 2011 for the six replicated trees and evaluated the effect of ‘year’ on biomass independently for each tree. We analyzed the measurements of reproductive biomass (total weight, seed weight, capsule weight, stem weight, cotton weight, and the weight of 100 seeds) using a linear mixed effects models following the procedures described in Pinhero and Bates (2000). In our analyses, genotype class and year were treated as fixed effects with ‘individual tree’ treated as a random effect. For all analyses we first fit a global model containing all the fixed and random effects. We fit an additive model because we were only interested in accounting for variation attributed to year and genotype class. The global model was then inspected for departures from normality and independence using plots of the residuals and quantile plots (qq-plots). We assessed the need for the random term in each model by inspecting the estimate and standard deviation of each of the random intercepts. If those intercepts were all bounded away from zero we kept the random effect in the model. When we detected departures from the assumption of homoscedasticity in the global model we fit a model with a heteroscedastic error term to see if the fit was improved (Pinheiro & Bates 2000). When we were satisfied with the fit of the global model we used ANOVA to determine if the fixed effects had a significant influence on response. If a significant fixed effect was detected we then examined the difference among the treatment means using Tukey’s Honestly Significant Difference (HSD) test.

We analyzed the measurements of reproductive yield (number of capsules, number of seeds and the number of seeds per capsule) in the same way as we analyzed the measurements of reproductive biomass. However, these data were counts so we fit the global model using a Poisson response rather than a Gaussian (normal) response (Table 2). The global model for the number of seeds per capsule was the same as the model for the number of seeds, but included an offset term for the number of capsules per catkin. This allowed us to model the effect of genotype and year on the number of seeds instead of first calculating a rate. Count data can suffer from overdispersion (i.e., more variability in the data than expected by the model) (McCullagh & Nelder 1983), therefore we assessed the global models for overdispersion before assessing them for departures from normality. We determined that data for the number of seeds and the number of seeds per capsule were overdispersed so we fit a Poisson-lognormal distribution by adding a random term to the model for each observation in the data (Elsto*n et a*l. 2001).

The results of the fungal inoculation experiment were analyzed as a linear mixed effect model. As before, we treated genotype class as a fixed effect and individual tree as a random effect. Year was not included as an explanatory factor in this experiment and each fungal inoculation experiment was analyzed independently. The data were counts of fungal uredia on the surface of leaves, therefore each model included an offset term for the size of the sampled leaf. We determined that the data from all three experiments were overdispersed so we fit a Poisson-lognormal distribution as with the analysis of reproductive yield. The raw data for these experiments contained many zero values (no uredia grew on the leaves) which led to issues with model fit. The data were also overdispersed (Suppl. File 3). These two issues were only partially corrected by fitting a log-Poisson model, therefore the parameter estimates derived from these models may be overly optimistic and the true influence of genotype class on fungal growth may be overestimated and the lack of statistical significance in some of the results may be due to poor model fit.

The method we used to fit the linear mixed effects models when applied to Poisson data does not produce traditional F and p values for the significance of the various factors (Bate*s et a*l. 2012). Therefore, to test the effect of year and genotype class on reproductive yield and the effect of genotype class on the number of fungal uredia we fit single factor models (i.e. models that just contained genotype or year) and a ‘degenerate’ model containing only the random effects and then compared the fit of these models to the global model using AIC, BIC and the log-likelihood chi-square test. If a significant fixed effect was detected then differences among the treatment means were analysed as before using Tukey’s HSD test.

The results of the seed germination trials (proportion successful germinants, proportion of abnormal germinates) were analyzed as general linear models following the procedures in Venables and Ripley (2002). As before, genotype class and year were treated as fixed effects with an additional fixed effect of TSW. In this experiment there was no need for a random effect as there was only a single estimate of yield for each tree in each year. Each model also included an offset term for the number of seeds that were tested in each germination trial. This allowed us to directly test the influence of the fixed effects on the number of seeds that germinated instead of the rate of germination. We fit the global model using a Poisson distribution, but the data were overdispersed so we fit a negative binomial which provided a better fit to the data (McCullagh & Nelder 1983; Venables & Ripley 2002). The negative binomial distribution is similar to the Poisson except that it includes a term that models the dispersion in the response (Venables & Ripley 2002). The influence of tree genotype on some tree characteristics (age, height and diameter at breast height) was also analyzed as a general linear model but we did not include year as an effect in these models. For both sets of analyses, the fit of the global model to the data was analyzed as before. Factors in the model were tested for significance by adding them sequentially and comparing the fit between adjacent models. This is similar to the procedure used to analyze the yield and inoculation data, except here F and p values can be produced. Differences among the levels of the fixed effects were analyzed using Tukey’s HSD.

**Data S3**: R code for analyses.

**Table S1 Summary of statistical methods used in analysis of poplar data. Fixed and random effects are given for the global model after testing for normality and overdisperssion. There were three levels of the genotype class fixed effect and two levels of the year fixed effect; 1 000 seed weight was continuous variable that was centred on the mean value before fitting the model. Offset terms, when present, were log transformed within the model. Abbreviations of fungal species as follows: Mlp: *Melampsora larici-populina*, Mmd: *M. medusae f. sp. deltoidae*, and Mo: *M. occidentalis***

| **Test** | **Response** | **Fixed effect(s)** | **Random effect(s)** | **Offset term** | **Distribution** |
| --- | --- | --- | --- | --- | --- |
| *Reproductive biomass* | |  |  |  |  |
|  | 100 seed | genotype, year | individual tree |  | Gaussian |
|  | capsule | genotype, year | individual tree |  | Gaussian |
|  | cotton | genotype, year | individual tree |  | Gaussian |
|  | seed | genotype, year | individual tree |  | Gaussian |
|  | stem | genotype, year | individual tree |  | Gaussian |
|  | total biomass | genotype, year | individual tree |  | Gaussian |
|  |  |  |  |  |  |
| *Reproductive yield* | |  |  |  |  |
|  | No. of capsules | genotype, year | tree |  | Poisson |
|  | No. of seeds | genotype, year | tree |  | Poisson |
|  | No. seeds per capsule | genotype, year | tree, observation | No. of capsules in the sampled catkin | Poisson lognormal |
|  | |  |  |  |  |
| *Seed Germination* | |  |  |  |  |
|  | Successful germinates | genotype, 1000 seed weight, year |  | No. of seeds tested | Negative binomial |
|  | Abnormal germinates | genotype, 1000 seed weight, year |  | No. of seeds tested | Negative binomial |
|  |  |  |  |  |  |
| *Disease susceptibility* | |  |  |  |  |
|  | No. of Mlp uredia | genotype | tree, observation | surface area of sampled leaf | Poisson lognormal |
|  | No. of Mmd uredia | genotype | tree, observation | surface area of sampled leaf | Poisson lognormal |
|  | No. of Mo uredia | genotype | tree, observation | surface area of sampled leaf | Poisson lognormal |
|  |  |  |  |  |  |
| *Stand characteristics* | |  |  |  | Gaussian |
|  | Diameter at breast height | genotype |  |  | Gaussian |
|  | Height | genotype |  |  | Gaussian |

**Table S2**: Distribution of age classes estimated for reproductively mature *P. balsamifera* (B), *P. deltoides* (D), and native hybrids (DxB) at BPSF.

|  |  | Estimated age (years) | | | |
| --- | --- | --- | --- | --- | --- |
|  | n | <20 | 20-40 | 40-60 | >60 |
| B | 14 | 5 | 9 | 0 | 0 |
| DxB | 14 | 1 | 11 | 1 | 1 |
| D | 100 | 7 | 42 | 28 | 23 |

**Table S3**: Summary of the linear mixed effects model analysis for the effect of year and genotype on six biomass measurements from catkins of *P. balsamifera* (B), *P. deltoides* (D), and native hybrids (D×B). Tukey’s Honestly Significant Difference test was used to identify differences among genotype class means when a significant effect was detected. A. Full data set; B. Partial data set is restricted to single measurements per tree.

| **A. Full** |  |  | | | **Post-hoc** | |  |
| --- | --- | --- | --- | --- | --- | --- | --- |
| Trait evaluated | factor | df | F | *p* | B | DxB | D |
| *Total biomass* | Intercept | 1,177 | 222.554 | <0.0001 |  |  |  |
|  | genotype | 2,18 | 6.417 | **0.0079**^†^ | a | b | ab |
|  | year | 1,18 | 2.216 | 0.154 |  |  |  |
|  |  |  |  |  |  |  |  |
| *Total seed* | Intercept | 1,177 | 93.888 | <0.0001 |  |  |  |
| *biomass* | genotype | 2,18 | 4.186 | **0.0322**^†^ | a | b | ab |
|  | year | 1,18 | 0.474 | 0.500 |  |  |  |
|  |  |  |  |  |  |  |  |
| *100- seed* | Intercept | 1,177 | 319.258 | <0.0001 |  |  |  |
| *weight* | genotype | 2,18 | 5.857 | **0.011** | a | a | b |
|  | year | 1,18 | 1.062 | 0.316 |  |  |  |
|  |  |  |  |  |  |  |  |
| *Capsule biomass* | Intercept | 1,177 | 235.415 | <0.0001 |  |  |  |
|  | genotype | 2,18 | 10.757 | **0.0008** | a | b | b |
|  | year | 1,18 | 0.652 | 0.430 |  |  |  |
|  |  |  |  |  |  |  |  |
| *Stem biomass* | Intercept | 1,177 | 139.647 | <0.0001 |  |  |  |
|  | genotype | 2,18 | 8.814 | **0.0021** | a | b^†^ | b |
|  | year | 1,18 | 0.113 | 0.740 |  |  |  |
|  |  |  |  |  |  |  |  |
| *Cotton biomass* | Intercept | 1,177 | 201.043 | <0.0001 |  |  |  |
|  | genotype | 2,18 | 2.451 | 0.1145 |  |  |  |
|  | year | 1,18 | 5.816 | **0.0268**^†^ |  |  |  |

| **B. Partial** |  |  | | | **Post-hoc** | |  |
| --- | --- | --- | --- | --- | --- | --- | --- |
| Traits evaluated | Factor | df | F | *p* | B | DxB | D |
| *Total biomass* | Intercept | 1,116 | 107.780 | <0.0001 |  |  |  |
|  | genotype | 2,10 | 2.719 | 0.114^†^ |  |  |  |
|  | year | 1,10 | 0.218 | 0.651 |  |  |  |
|  |  |  |  |  |  |  |  |
| *Total seed* | Intercept | 1,116 | 37.663 | <0.0001 |  |  |  |
| *biomass* | genotype | 2,10 | 1.886 | 0.202^†^ |  |  |  |
|  | year | 1,10 | 0.0420 | 0.842 |  |  |  |
|  |  |  |  |  |  |  |  |
| *100 seed* | Intercept | 1,116 | 203.031 | <0.0001 |  |  |  |
| *biomass* | genotype | 2,10 | 7.620 | **0.0098** | a | a | b |
|  | year | 1,10 | 2.498 | 0.145 |  |  |  |
|  |  |  |  |  |  |  |  |
| *Capsule* | Intercept | 1,116 | 135.509 | <0.0001 |  |  |  |
| *biomass* | genotype | 2,10 | 6.171 | **0.018** | a | b | b |
|  | year | 1,10 | 0.000320 | 0.986 |  |  |  |
|  |  |  |  |  |  |  |  |
| *Stem biomass* | Intercept | 1,116 | 202.941 | <0.0001 |  |  |  |
|  | genotype | 2,10 | 5.004 | **0.0312** | a | b | ab^†^ |
|  | year | 1,10 | 1.614 | 0.233 |  |  |  |
|  |  |  |  |  |  |  |  |
| *Cotton biomass* | Intercept | 1,116 | 88.311 | <0.0001 |  |  |  |
|  | genotype | 2,10 | 1.113 | 0.366 |  |  |  |
|  | year | 1,10 | 2.064 | 0.181^†^ |  |  |  |

^†^Differs in full data set

**Table S4**: Summary of the results of the linear mixed effects model analysis of reproductive yield of *P. balsamifera* (B), *P. deltoides* (D), and native hybrids (DxB). Both tree class and year were treated as fixed effects. Tukey’s Honestly Significant Difference test was used to identify differences among tree class means when a significant effect was detected.

| **A. Full** |  |  |  |  |  |  |  |  | **Post-hoc** | | |
| --- | --- | --- | --- | --- | --- | --- | --- | --- | --- | --- | --- |
| response | Predictors | df | AIC | BIC | Log-Likelihood | χ^2^ | χ^2^ df | *p* | B | DxB | D |
| *No. of capsules* | null* | 2 | 213.64 | 220.07 | -104.820 |  |  |  |  |  |  |
| *per catkin* | year | 3 | 215.26 | 224.91 | -104.631 | 0.379 | 1 | 0.538 |  |  |  |
|  | genotype | 4 | 188.96 | 201.82 | -90.482 | 28.298 | 1 | **<0.0001** | a | b | c |
|  | year + genotype | 5 | 189.36 | 205.44 | -89.680 | 1.6039 | 1 | 0.2054 |  |  |  |
|  |  |  |  |  |  |  |  |  |  |  |  |
| *No. of seeds* | null* | 3 | 783.99 | 793.63 | -388.99 |  |  |  |  |  |  |
| *per capsule* | year | 4 | 782.32 | 795.18 | -387.16 | 3.666 | 1 | 0.0555 |  |  |  |
|  | genotype | 5 | 779.84 | 795.91 | -384.92 | 4.483 | 1 | **0.0342**^†^ | ab | a | b |
|  | year + genotype | 6 | 774.81 | 794.10 | -381.41 | 7.027 | 1 | **0.00803**^†^ |  |  |  |
|  |  |  |  |  |  |  |  |  |  |  |  |
| *No. of seeds* | null* | 3 | 934.31 | 944.19 | -464.16 |  |  |  |  |  |  |
| *per catkin* | year | 4 | 934.77 | 947.95 | -463.39 | 1.539 | 1 | 0.215 |  |  |  |
|  | genotype | 5 | 933.26 | 949.73 | -461.63 | 3.509 | 1 | 0.0610 |  |  |  |
|  | year + genotype | 6 | 932.32 | 952.08 | -460.16 | 2.947 | 1 | 0.0861 |  |  |  |

| **B. Partial** |  |  |  |  |  |  |  |  | Post-hoc | | |
| --- | --- | --- | --- | --- | --- | --- | --- | --- | --- | --- | --- |
| Response | Predictors | df | AIC | BIC | Log-Likelihood | χ^2^ | χ^2^ df | P | B | DxB | D |
| *No. of capsules* | null* | 2 | 148.09 | 153.67 | -72.047 |  |  |  |  |  |  |
| *per catkin* | year | 3 | 149.89 | 158.25 | -71.946 | 0.202 | 1 | 0.653 |  |  |  |
|  | genotype | 4 | 131.26 | 142.41 | -61.629 | 20.634 | 1 | **<0.0001** | a | b | c |
|  | year + genotype | 5 | 132.02 | 145.96 | -61.010 | 1.237 | 1 | 0.266 |  |  |  |
|  |  |  |  |  |  |  |  |  |  |  |  |
| *No. of seeds* | null* | 3 | 501.580 | 509.940 | -247.790 |  |  |  |  |  |  |
| *per capsule* | year | 4 | 500.310 | 511.460 | -246.150 | 3.274 | 1 | 0.0704 |  |  |  |
|  | genotype | 5 | 502.41 | 516.35 | -246.21 | 0.000 | 1 | 1.0000 |  |  |  |
|  | year + genotype | 6 | 496.41 | 513.13 | -242.20 | 8.006 | 1 | **0.00466**^†^ | a | b | a |
|  |  |  |  |  |  |  |  |  |  |  |  |
| *No. of seeds* | null* | 3 | 607.84 | 616.44 | -300.92 |  |  |  |  |  |  |
| *per catkin* | year | 4 | 609.24 | 620.71 | -300.62 | 0.595 | 1 | 0.440 |  |  |  |
|  | genotype | 5 | 609.03 | 623.37 | -299.52 | 2.209 | 1 | 0.137 |  |  |  |
|  | year + genotype | 6 | 609.54 | 626.74 | -298.77 | 1.499 | 1 | 0.221 |  |  |  |

* random effects only ^†^ Significance differs in full and partial data sets

**Table S5**: Summary of the general linear model analysis for the effect of genotype and 1000 seed weight (TSW) on seed germination for seeds from *P. balsamifera* (B), pure *P. deltoides* (D), and native hybrids (D×B). Tukey’s Honestly Significant Difference test was used to identify differences among genotype class means when a significant effect was detected. In 2009 we tested TSW, and genotype class; while in 2009-2011 we excluded multiple sampled trees to create a balanced data set in order to test the additional effect of year (see Suppl. File 2 for detailed description of rationale).

|  |  |  |  |  |  |  |  | **Post-hoc** | | |
| --- | --- | --- | --- | --- | --- | --- | --- | --- | --- | --- |
| Test | Factor | df | Deviance explained | Residual  df | Residual deviance | *p* ( *χ*^2^ ) | F^†^ | B | DxB | D |
| *2009* | |  |  |  |  |  |  |  |  |  |
| *Successful germination* | |  |  |  |  |  |  |  |  |  |
| null | |  |  | 26 | 38.561 |  |  |  |  |  |
| TSW | | 1 | 0.708 | 25 | 37.853 | 0.400 | 0.708 |  |  |  |
| tree class | | 2 | 9.643 | 23 | 28.209 | **0.00805** | 4.822 | a | a | b |
| *Abnormal germination* | |  |  |  |  |  |  |  |  |  |
| null | |  |  | 26 | 64.892 |  |  |  |  |  |
| TSW | | 1 | 2.118 | 25 | 62.775 | 0.146 | 2.118 |  |  |  |
| tree class | | 2 | 33.497 | 23 | 29.277 | **<0.0001** | 16.749 | a | b | c |
| *2009 - 2011* | |  |  |  |  |  |  |  |  |  |
| *Successful germination* | |  |  |  |  |  |  |  |  |  |
|  | null |  |  | 34 | 70.833 |  |  |  |  |  |
|  | TSW | 1 | 0.0600 | 33 | 70.774 | 0.8073 | 0.0595 |  |  |  |
|  | tree class | 2 | 32.878 | 31 | 37.896 | **<0.0001** | 16.439 | a | a | b |
|  | year | 2 | 0.488 | 29 | 37.409 | 0.7836 | 0.244 |  |  |  |
| *Abnormal germination* | |  |  |  |  |  |  |  |  |  |
|  | null |  |  | 34 | 75.024 |  |  |  |  |  |
|  | TSW | 1 | 0.3755 | 33 | 74.649 | 0.540 | 0.376 |  |  |  |
|  | tree class | 2 | 31.1653 | 31 | 43.483 | **<0.0001** | 15.583 | a | a | b |
|  | year | 2 | 3.3109 | 29 | 40.173 | 0.191 | 1.656 |  |  |  |

^†^ F values are included for convenience. They should be interpreted with caution as the dispersion in these models is not constant.

**Table S6**: Summary of the linear mixed effects model analysis for the effect of genotype on infection severity (uredia) of poplar rust on *P. balsamifera* (B), *P. deltoides* (D), and native hybrids (D×B). Results are based on controlled inoculations of *Melampsora larici-populina* (*Mlp*), *M. medusae f.sp. deltoidae* (*Mmd*), and *M. occidentalis* (*Mo*). Tukey’s Honestly Significant Difference test was used to identify differences among tree genotype means when a significant fixed effect was detected.

|  |  |  |  |  |  |  |  |  | **Post-hoc** | | |
| --- | --- | --- | --- | --- | --- | --- | --- | --- | --- | --- | --- |
| Fungus | Model | df | AIC | BIC | Log-Liklihood | *Χ^2^* | df | *p* | B | DxB | D |
| *Mlp* | 1 | 3 | 178.130 | 184.470 | -86.067 |  |  |  |  |  |  |
|  | 2 | 5 | 160.360 | 170.920 | -75.181 | 21.772 | 2 | **<0.001** | a | a | b |
|  |  |  |  |  |  |  |  |  |  |  |  |
| *Mmd* | 1 | 3 | 254.280 | 260.990 | -124.140 |  |  |  |  |  |  |
|  | 2 | 5 | 229.480 | 240.660 | -109.740 | 28.800 | 2 | **<0.001** | a | a | b |
|  |  |  |  |  |  |  |  |  |  |  |  |
| *Mo* | 1 | 2 | 353.340 | 358.070 | -174.670 |  |  |  |  |  |  |
|  | 2 | 4 | 334.280 | 343.760 | -163.140 | 23.058 | 2 | **<0.001** | a | b | ab |

Model 1: number of uredia ~ random effects (tree, observations per tree)

Model 2: number of uredia ~ tree class + random effects

**Table S7**: Field surveys of fungal disease at Base de plein-air de Sainte-Foy. *Melampsora* spp. incidence was recorded as one of three damage classes: absent, <50% leaf area, and >50% leaf area. *Septoria* spp. leaf spot was recorded as presence or absence. Proportion of observed trees in each class is shown in brackets.

|  | *Melampsora* spp. | | | |  | *Septoria* spp. | | |
| --- | --- | --- | --- | --- | --- | --- | --- | --- |
| tree class | n | absent | <50% | >50% |  | n | absent | present |
| *P. balsamifera* | 7 | 2 (0.29) | 3 (0.43) | 2 (0.29) |  | 8 | 0 | 8 (1.00) |
| DxB | 8 | 3 (0.38) | 3 (0.38) | 2 (0.25) |  | 11 | 0 | 11 (1.00) |
| *P. deltoides* | 64 | 30 (0.47) | 20 (0.31) | 14 (0.22) |  | 68 | 4 (0.06) | 64 (0.94) |
