# Supplementary figures and images for: Fitness dynamics within a poplar hybrid zone: I. Prezygotic and postzygotic barriers impacting a native poplar hybrid stand
Source: Ecol Evol. 2014 Apr 3;4(9):1629–47. doi: 10.1002/ece3.1029 (PMC4063464; doi:10.1002/ece3.1029)

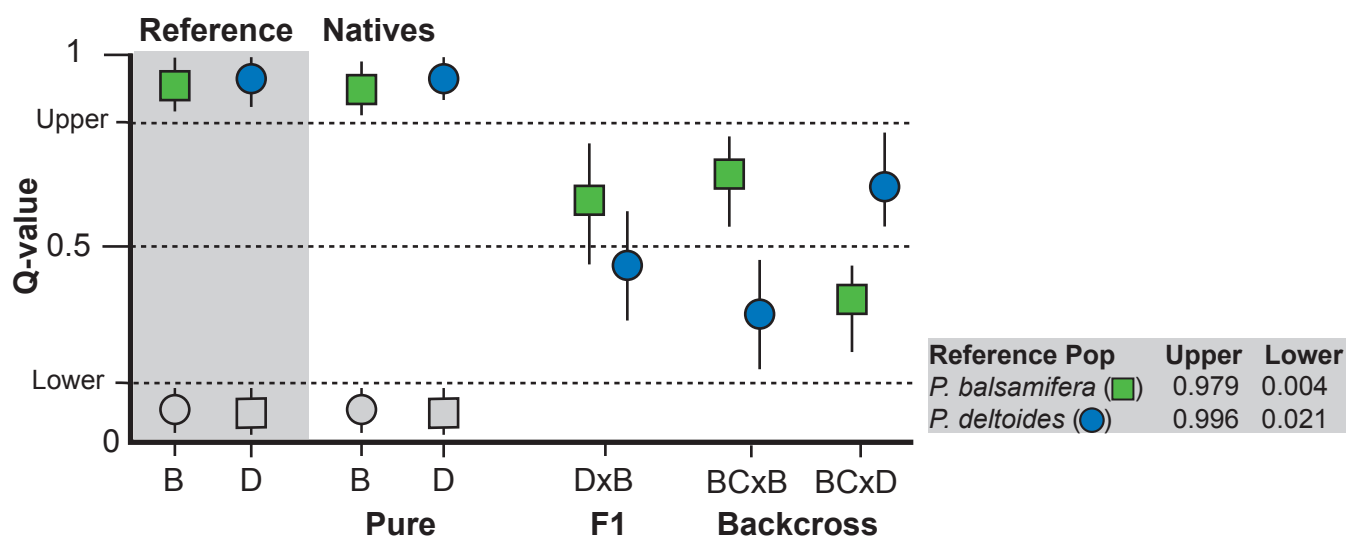

**Supplemental File 1**

Supplement: Supplementary file 2 [file ece30004-1629-SD2.pdf]
